# Reproducing RECIST lesion selection via machine learning: Insights into intra and inter-radiologist variation

**DOI:** 10.1016/j.ejro.2024.100562

**Published:** 2024-04-17

**Authors:** Teresa M. Tareco Bucho, Liliana Petrychenko, Mohamed A. Abdelatty, Nino Bogveradze, Zuhir Bodalal, Regina G.H. Beets-Tan, Stefano Trebeschi

**Affiliations:** a Department of Radiology, Netherlands Cancer Institute, Amsterdam, the Netherlands; b GROW School for Oncology and Reproduction, Maastricht University, Maastricht, the Netherlands; c Department of Radiology, Kasr Al Ainy Hospital, Cairo University, Cairo, Egypt; d Department of Radiology, American Hospital Tbilisi, Tbilisi, Georgia; e Faculty of Health Sciences, University of Southern Denmark, Denmark

**Keywords:** RECIST, Cancer imaging, Machine learning, Variability, Reproducibility

## Abstract

**Background:**

The Response Evaluation Criteria in Solid Tumors (RECIST) aims to provide a standardized approach to assess treatment response in solid tumors. However, discrepancies in the selection of measurable and target lesions among radiologists using these criteria pose a significant limitation to their reproducibility and accuracy. This study aimed to understand the factors contributing to this variability.

**Methods:**

Machine learning models were used to replicate, in parallel, the selection process of measurable and target lesions by two radiologists in a cohort of 40 patients from an internal pan-cancer dataset. The models were trained on lesion characteristics such as size, shape, texture, rank, and proximity to other lesions. Ablation experiments were conducted to evaluate the impact of lesion diameter, volume, and rank on the selection process.

**Results:**

The models successfully reproduced the selection of measurable lesions, relying primarily on size-related features. Similarly, the models reproduced target lesion selection, relying mostly on lesion rank. Beyond these features, the importance placed by different radiologists on different visual characteristics can vary, specifically when choosing target lesions. Worth noting that substantial variability was still observed between radiologists in both measurable and target lesion selection.

**Conclusions:**

Despite the successful replication of lesion selection, our results still revealed significant inter-radiologist disagreement. This underscores the necessity for more precise guidelines to standardize lesion selection processes and minimize reliance on individual interpretation and experience as a means to bridge existing ambiguities.

## 1 Introduction

The Response Evaluation Criteria in Solid Tumors (RECIST) is a widely adopted standardized approach for assessing the response of solid tumors to treatment. A crucial aspect of RECIST involves the identification of measurable lesions and the subsequent selection of target lesions. Target lesions are a subset of lesions chosen to represent the overall response of the total tumor burden to treatment. RECIST guidelines provide specific criteria for identifying measurable and target lesions, to the detriment of others. For instance, in RECIST version 1.1, measurable lesions must have a minimum diameter of 10 mm, except for lymph nodes, which require a short-axis measurement of at least 15 mm [4]. Among the measurable lesions, up to five target lesions can be selected in total, with a maximum of two per organ. When faced with more than five eligible lesions, RECIST advises choosing those with the largest diameter and those that “lend themselves to reproducible repeated measurements” [4]. The selection of target lesions is key, as it is primarily based on the diameter measurements of these lesions that the patient’s response to treatment is determined.

Despite RECIST's goal of establishing a standardized framework for evaluating tumor response and allowing comparisons of treatment efficacy across different centers and clinical trials, several limitations hinder its accuracy and reproducibility. These limitations stem from various factors, including errors in measuring unidimensional diameters [18], discrepancies in the detection of new lesions [1], and inter-reader variability due to differences in experience [16]. One significant limitation, which is the central focus of this study and prior research [10], [7], [8], pertains to the selection of target lesions. The requirement to choose a limited set of up to five lesions demands the prioritization of certain lesions over others, thereby excluding a substantial number of lesions from quantitative assessments in metastatic settings.

Despite knowing that different radiologists often select different target lesions and that these lesions may exhibit diverse responses to treatment, resulting in conflicting overall response outcomes, the underlying reasons for discrepancies in lesion selection among radiologists with comparable experience levels remain unexplored. To elucidate the factors influencing radiologists' decisions in choosing one lesion over another, we designed an experiment to replicate the lesion selection process. Specifically, we employed machine learning models trained on lesion characteristics to predict which lesions were measurable and/or target lesions, and. assessed the importance of these features in the selection process. These features may represent practical considerations in the selection process, such as ease of measurement, or biological activity, such as lesion aggressiveness. An additional reader is then introduced and a parallel analysis is performed to understand the differences in lesion selection between radiologists.

## 2 Materials and methods

### 2.1 Study cohort

In this study, a retrospective selection of patients was conducted from the PAN-CANCER study, currently unpublished (ethical approval IRB-d19-147). This comprehensive study includes a substantial cohort of cancer patients, and is characterized by matched radiological imaging with biopsy data. The inclusion criteria for this research were specifically designed to encompass all patients admitted to our institute on or before 2021, who underwent a contrast-enhanced high-resolution CT scan within a 90-day period prior to a biopsy involving next-generation sequencing. Exclusions were made for scans that demonstrated severe imaging artifacts or where no discernible lesions were present (e.g., postoperative cases). Subsequently, a specialized team of radiologists was tasked with the identification and delineation of all malignant lesions, adhering to a maximum limit of ten lesions per organ. Detailed criteria applied to the segmentation process are thoroughly outlined in Supplement 1.

To remove the impact of differences in lesion delineation among radiologists, our selection criteria were restricted to include patients whose lesions were segmented by a specific radiologist (NB, two years of CT reading experience). Additionally, we included all instances where every measurable lesion was precisely segmented, while excluding cases with incidental findings such as overlooked or mischaracterized lesions, or benign manifestations. A secondary review was conducted by another radiologist (LP, with four years of CT reading experience) to further validate the findings. Only cases without any incidental findings were chosen for inclusion in our study. This step ensured that our dataset was composed exclusively of patients with comprehensive segmentations of all detectable tumor lesions.

### 2.2 Measurable and target lesion selection

An experienced radiologist (LP, reader 1, four years of experience) was tasked to review each CT scan and classify all lesions as either measurable or non-measurable and, among the measurable ones, to choose the target lesions according to RECIST 1.1 criteria. A second reader (MA, reader 2, six years of experience) was given the same tasks, blinded to the assessment of reader 1. To compel the radiologists to decide between RECIST target lesions and other measurable lesions, the dataset was further refined to include only what we have termed "actionable scans": scans of patients with either three or more lesions in a single organ, or more than five lesions in total. Such criteria helped focus the analysis on the process of decision making, where a clear choice between different types of lesions was necessary. The image analysis for these scans was conducted using the 3D Slicer tool (www.slicer.org).

### 2.3 Replicating lesion selection by machine learning

To simulate the selection of target lesions, we utilized a random forest classifier. This classifier was fed with lesion characteristics (radiomics features) describing shape, size and texture, extracted using PyRadiomics version 3.0.1 [17]; contextual information, including organ location, lesion's rank in size within the organ (i.e. largest, second largest,.: organ rank) and across the body (overall rank), as well as proximity to other lesions. A comprehensive description of the included features can be found in Supplement 2.

**Fig. 1:**
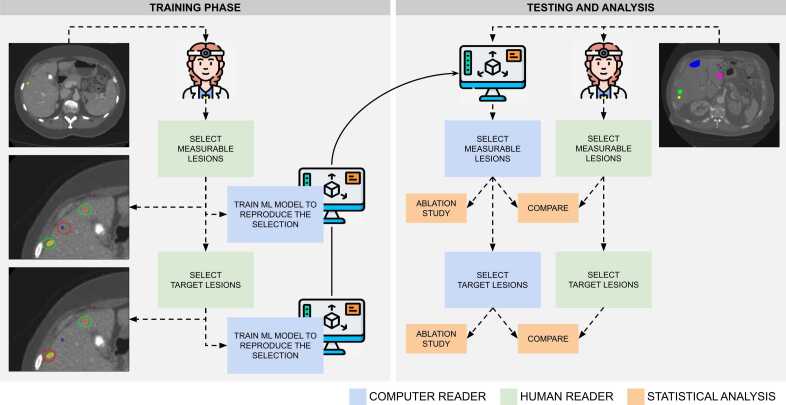
Schematic representation of the data analysis. Icons from flaticon.com.

Next, the classifier models were trained using the lesion selection made by a single radiologist (reader 1) to determine whether a lesion would be categorized as measurable/non-measurable and target/non-target. By examining the mean decrease in impurity for each feature, we assessed the importance of the features in the decision-making process of the model. This analysis allowed us to gain insights into the characteristics of the lesions that drive radiologists to select a lesion as measurable or target. The details of the models’ training code can be found in Supplement 3. The procedure was repeated independently for reader 2, resulting in a second model. Consequently, through this procedure, we developed two distinct models, one for each reader, aiming to replicate their respective lesion selection procedures. Fig. 1 shows a schematic of the procedure. To investigate the relationship between the decision-making criteria driving each radiologist to make their lesion selection, we compute the correlation between the most important features of each reader’s model.

### 2.4 Impact of diameter, volume, and size rank on lesion selection

Considering that the sizeof a lesion, as per the RECIST criteria, holds significant importance in determining its measurability and, more importantly, its suitability as a target lesion, we conducted three ablation experiments. “Ablation” in this study refers to the machine learning practice of studying the importance of a component or a feature by observing the change of performance of a model after removing or “ablating” it. Firstly, we ablated 2D axial diameter, and all features that showed a correlation coefficient above a threshold of ρ*=*0.5 with the 2D axial diameter, while retaining overall rank, organ rank (which are based on diameter), and volume as features. Secondly, we removed both the 2D axial diameter and volume, along with any features correlated with them, and retained only rank features. Lastly, we removed lesion ranks (overall and within the organs) and correlated features. These experiments allow us to explore the extent to which tumor size contributes to the selection process of the radiologist.

### 2.5 Validation and statistical analysis

Patients were divided into a train (70 %) and a test (30 %) fold. Due to the small size of the dataset, this procedure was repeated 100 times by shuffling the dataset before splitting, in a random and repeatable procedure called Monte-Carlo cross-validation approach (MCCV) [11], to obtain a stable performance estimate with reduced uncertainty. For each of the splits, the training folds were used to build the model and then its performance was assessed on the independent test fold. All models were trained and tested on the same train-test splits to ensure the comparability of the results. The area under the receiver operating characteristic curve (ROC-AUC) was computed to compare the performance of the models. We report both the overall ROC-AUC (across all lesions in the test set) and mean ROC-AUC on a patient level. When all lesions of a patient belonged only to one class (e.g., all lesions were considered measurable), then it is not possible to compute the AUC, and these patients were excluded from the patient-level and statistical analysis. Non-parametric paired permutation tests (two-sided) [13] were employed to compare the median ROC-AUC, with N=10.000 resamples, implemented in the SciPy library version 1.10.1. P-values were corrected with Bonferroni when appropriate. A p-value below.05/c was considered as significant, where c is the number of comparisons. The Mann-Whitney U test was used to compare lesion sizes. To analyze the correlation between feature importances for both readers, the Spearman correlation was used. The utilization of Cohen's Kappa was precluded in our analysis to assess agreement due to the dependence between lesions belonging to the same patient and during target lesion selection; specifically, the selection of one lesion could potentially influence the selection of subsequent lesions within the same patient.

## 3 Results

In total, we retrieved N=582 scans segmented by the same radiologist. Of them, N=177 were eligible as “actionable”, i.e. where the radiologist had the option to choose lesions: having at least one organ with three lesions, or more than five in total, and at most nine lesions in all organs involved. Of them, N=40 scans (1 scan per patient) were included in the study by the second radiologist for meeting the required quality inclusion criteria (see Section 2.1). There were a total of 263 segmented lesions, the majority of which located in the liver (N=88), mediastinal lymph nodes (N=42), lung (N=40) and abdomen (N=34). A complete list of the number of lesions per organ can be found in Supplement 4.

Each patient had a median of 6.5 lesions (95 % CI [3−11]), three per organ, with a median of two organs involved. Lesions were, on average, 17.7 mm in diameter (CI [6.9–67.4] mm) and 1.6 mm^3^ in volume (CI [0.07–91.7] mm^3^). Lung cancer (N=18) was the most common cancer type, followed by colorectal (N=8). The average age was 66.0 (CI [37.8–77.3]) years and 47.5 % were females. All CT scans were diagnostic scans with contrast enhancement and slice thickness <5 mm.

### 3.1 Can we replicate the selection of lesions with machine learning?

The models reached an overall median ROC-AUC of 0.82 in replicating the selection of measurable lesions and 0.79 for target lesions. On a patient level, median ROC-AUC was 0.88 and 0.87 for measurable and target selection respectively. Analysis of the median feature importances of the models revealed that surface volume ratio, minor axis length, and volume were the most important features for measurable lesions selection, and organ rank, overall rank, and volume for target lesion selection In Supplement 5, the top five features for all experiments are listed.

### 3.2 How does lesion diameter, volume, and rank impact the selection process?

**Fig. 2:**
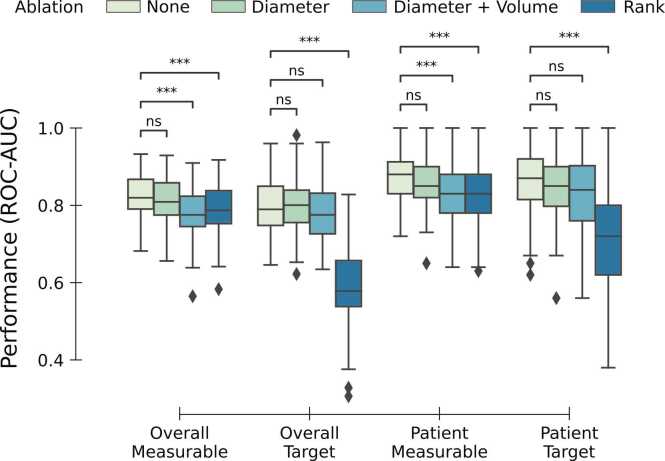
Performance of the models in measurable and target lesion selection of the models across the 100 MCCV test folds, on an overall and patient-level, for the across different ablation experiments and settings. Nn: not-significant; ***: p-value < 0.001.

To quantify the impact of lesion diameter, rank, and volume on the selection process, we performed three ablation experiments, where we blinded each model to diameter, rank, and volume information by removing this feature and correlated ones (**ρ** > 0.5) from the set of input features. Blinding the models to diameter information resulted in an overall drop of performance: in measurable lesion selection from 0.82 to 0.81 (p=0.65) and in target lesion selection from 0.79 to 0.80 ROC-AUC (p=0.86). A similar pattern was observed on a patient level, where the median ROC-AUC decreased from 0.88 to 0.85 (p=0.051) for measurable lesions, and, for target lesions, from 0.87 to 0.85 (p=1.0). Removing volume, diameter and correlated features resulted in a further decline in overall performance: decrease from a median ROC-AUC of 0.82–0.78 (p<0.001) for measurable lesion selection, and, for target lesions selection, from 0.79 to 0.78 (p=1.0). A similar pattern was observed on a patient level, where the median ROC-AUC decreased from 0.88 to 0.83 (p<0.001) for measurable lesion prediction, and, for target lesion selection, from 0.87 to 0.84 (p=0.07). When removing rank and correlated features, the median ROC-AUC dropped from 0.82 to 0.79 (p<0.001) for measurable lesion selection and from 0.79 to 0.58 for target lesions (p<0.001). On a patient level, the performance dropped from 0.88 to 0.83 (p<=0.001) for measurable lesions and from 0.87 to 0.72 for target lesions (p<0.001). Fig. 2 shows an overview of the performance of the models for all ablation experiments.

### 3.3 Replication of the results with a second, independent reader

We replicated the analysis with a second, independent reader (six years of experience vs four years of the first reader), performing measurable and target lesion selection, blinded to the results of the first reader.

**Fig. 3:**
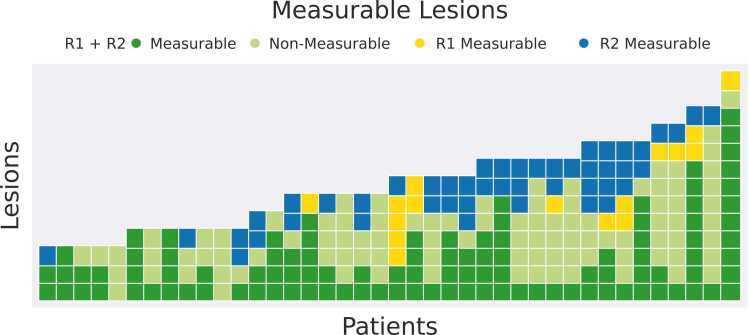
Overview of the dataset with the measurable lesions labeling from the readers. Color encodes the agreement between readers: green in case of agreement, yellow for lesions selected by reader 1, blue for lesions selected by reader 2.

We observed a high discordance in the selection of measurable lesions. Overall, the two readers agreed on identifying the same set of measurable lesions in 12 out of the 40 patients (30 %), including two patients with only non-measurable lesions and three patients with all measurable lesions. The median total number of lesions for concordant patients was 4, while for discordant patients, it was 8. An overview of the measurable lesion selection by the two readers can be seen in Fig. 3.

Discordant lesions (N=63) were distributed mostly in the liver (32 %), in the abdomen (21 %), and bone (N = 11 %). Lesions that were not selected as measurable by any of the readers (N=95) consisted mostly of mediastinal lymph nodes (32 %). Concordant measurable lesions (N=105) were distributed mostly in the liver (46 %), lung (22 %) and abdomen (15 %). Concordant measurable lesions were significantly larger (median diameter: 25.9 mm) than both concordant non-measurable (median diameter: 11.3 mm; p<=0.001) and discordant (median diameter: 16.4 mm; p<=0.001) lesions. In the comment section, available to the readers, a frequent explanation for not considering lesions as measurable was their failure to meet the minimum required diameter. This was observed even for discordant lesions, where one reader believed it was measurable while the other did not.

With respect to target lesions, readers agreed on selecting the same set of target/non-target lesions in 16 out of the 40 patients (40 %), of which five had disagreement in the preceding selection of measurable lesions. Eleven out of the 12 patients for which there was complete concordance on measurable lesions, had absolute agreement on target lesion selection.

**Fig. 4:**
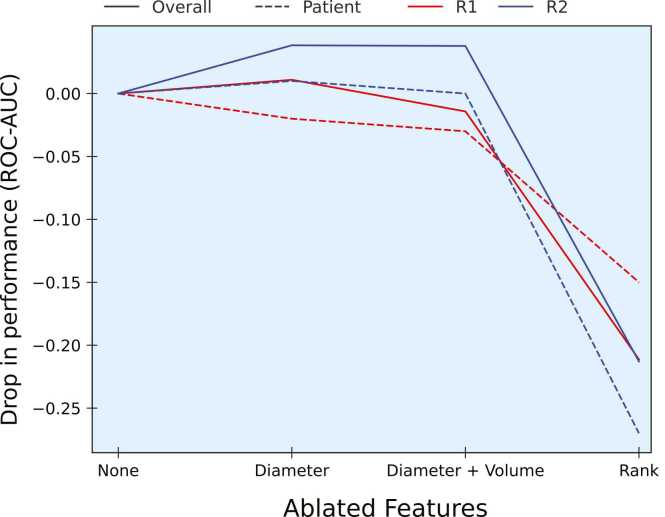
Comparative analysis of the models’ performances in the three ablation experiments between readers for target lesion selection.

When restricting the analysis to only the lesions that were considered measurable by at least one of the readers, readers agreed on 14 out of 38 patients (37.8 %). For simplicity, henceforth all our target lesion analyses will refer to this latter group of lesions, i.e. lesions that were classified as measurable by at least one reader. There were no patients with all target or all non-target lesions. The patients with concordant target lesion selection (both target and non-target) had a median total of three lesions distributed in one organ (median) and a median of two target lesions. The patients with discordant target lesion selection had a median total of 4.5 lesions, distributed in two organs (median) and a median of two target lesions. In comparison, patients with discordant target lesion selection had more lesions overall (4.5 lesions in median per patient), spread across more organs (two organs, median), and the same median number of target lesions (two) selected. Overall, concordant target lesions (N=69) were significantly larger (median diameter: 31.7 mm; p<=0.001) than both concordant non-target (N=44; median diameter: 15.8 mm; p<=0.001) and discordant (N= 55; median diameter: 18.8 mm) lesions. In regards to model performance, when comparing the first and second readers we observe a similar pattern in the performance in the ablation experiments. Particularly, the most pronounced reduction in performance for both readers occurred in the rank ablation experiment for target lesion selection (Fig. 4). The performance of the models for reader 2 across different ablation experiments can be found in Supplement 6.

**Fig. 5:**
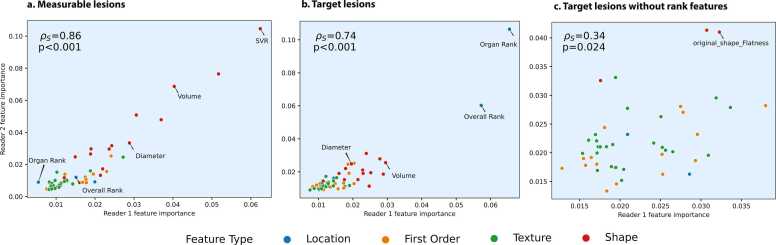
Correlation of the feature importance between reader 1 and reader 2 models, for the selection of (A) measurable lesions, (B) target lesions, and (C) target lesions with rank ablation.

When no ablation was performed, the most important features in measurable lesion selection were similar to those of reader 1: surface volume ratio, minor axis length and voxel volume; for target lesion selection these were overall rank, organ rank and major axis length (Supplement 5). Furthermore, we observed a strong correlation between the features’ importances of both readers for measurable (ρ=0.86, p<=0.001) and target lesion selection (ρ=0.74, p<=0.001, Fig. 5A). Given the large influence of organ rank and overall rank on the target lesion decision-making process (Fig. 5B), we additionally plotted the feature importances of both readers for the ablation experiment of rank features in target lesion selection(Fig. 5C) and observe a drop in the correlation (ρ=0.44, p=0.03), suggesting a lack of agreement of the features considered by the two readers.

## 4 Discussion

This study aimed to investigate the factors involved in the selection process of measurable and target lesions for response evaluation according to RECIST criteria by means of machine learning modeling. According to the guidelines, lesions are chosen based on size (minimum diameter for measurable lesions; largest lesions have priority as target), location (e.g. maximum two per organ), and visual examination (should be accurately measurable; should “lend themselves to reproducible repeated measurements”).

Following our results, it is possible to predict the selection of measurable lesions of individual radiologists by means of machine learning models. We observed that, in order to reproduce measurable lesion selection, the models relied mainly on shape features describing lesion size (minor axis length, volume) and shape (surface volume ratio). This is also confirmed by the ablation experiments, where the removal of volume causes the largest drop in performance. While this does not reflect exactly RECIST guidelines [4], it does allude to the size requirements, where measurable lesions are defined as lesions that can be accurately imaged and measured in at least one dimension in the axial plane in a CT scan, and have a minimum diameter of 10 mm, or 15 mm in the short axis for lymph nodes. All remaining requirements for measurable lesions do not rely on size: non-solid lesions, lesions in hollow organs, bone lesions without soft tissue components or previously irradiated lesions should not be considered as measurable. Thus, except for the minimum diameter criteria, measurable lesion selection should not be further influenced by their size if all other characteristics are similar: even small lesions can potentially be selected as measurable if their diameters surpass the minimum. The substantial drop in performance observed when removing volume and rank (defined based on diameter) suggests and reinforces the possibility of readers relying largely on the overall dimensions of a lesion compared to other lesions when deciding if it is measurable. It is possible that this a result of readers anticipating the upcoming task of selecting target lesions, which are chosen primarily based on their size — a phenomenon known as prospective memory: “remember to carry out an action that has been planned for a predefined time in the future, while performing a concurrent activity named ongoing task” [12], [3]. Nevertheless, it is important to observe that, despite the drop in performance caused by removing rank and size-related features, the models could still achieve good performance (AUC > 0.78), indicating that there are other characteristics of the lesions unrelated to their rank or size that are also important during the selection procedure.

Interestingly, while machine learning models could reproduce measurable lesion selection for each individual radiologist, we observed a substantial difference in the selection of measurable lesions between radiologists, which is unexpected to occur to such an extent, given the apparent objectiveness of the guidelines. Upon examining the possible causes of this disagreement, we observed that measurable lesions that were discordant had a significantly smaller size compared to concordant ones and that disagreement occurred to a large degree when the lesions’ diameter was close to the threshold of measurability (10 or 15 mm). The relationship between measurement precision and the surface area-to-volume ratio is inverse [5]. Therefore, the smaller the lesion, the less accurate its measurement is compared to larger lesions, which can justify higher disagreement between readers for smaller lesions. Independently of the size of the lesion, disagreement can also result from readers choosing different sections of the same lesion on which to measure the largest diameter [1], or different visualization settings (an example was documented by Beaumont et al. [1]), or questioning whether a lesion can be accurately measured all in all, e.g., due to unclear borders. A second relevant observation was the consistently larger selection in the number of measurable lesions by one radiologist compared to the other. Given these observations, one could hypothesize that the variability in the selection of measurable lesions could be attributed to radiologists relying on their own experience in cases where the guidelines are ambiguous. In other words, in situations where it is not clear how to measure a lesion, the radiologist may rely on their own experience, which will vary between radiologists, resulting in variability of measurements and, consequently, lesion selection. This phenomenon, where radiologists have their own reading pattern, dictated by differing experience, background, and interpretation of the guidelines, has been observed before by Iannessi and Beumount [6] in target lesion selection.

The strong correlation found between the features’ importances of the models of the two readers appears to challenge this point, suggesting similar patterns in measurable lesion selection. This observation could still be valid when considering that most of the disagreement will happen around cutoff values, where lesion size fails to provide a reproducible answer, while in general, the trend for larger lesions remains the same. There is a limited amount of research available on the variability of measurable lesion selection: Iannessi and Beaumont [6] found a noticeable discrepancy in differentiating between measurable and non-measurable disease, which was suggested to be linked to the small tumor burden.

Not accurately determining measurable disease can have implications in the subsequent stage of determining target lesions, particularly in the assessment of the non-target ones. While there are criteria to guide the selection of target lesions, non-target lesions simply consist of the remainder of the measurable lesions. As a result, any uncertainties regarding the measurability of lesions are likely to introduce variability primarily in non-target lesions [14]. Even though the assessment of non-target disease is primarily qualitative during response evaluation, non-target progression is not as rare as expected [2] and can still impact the overall outcome of the response assessment.

Similar to measurable lesions, our results suggest that it is possible to predict the selection of target lesions according to an individual radiologist. In the selection procedure, the models largely accounted for lesion size rank, both within the organ as well as per patient, to decide which lesion is a target, which is supported by the drop in performance in the models when rank-related features are removed. This aligns with the current RECIST guidelines, where the largest lesions per organ should be selected as a target. The guidelines for the selection of target lesions are comparatively more permissive than for measurable lesions, as radiologists can choose which organs to take the target lesions from, as well as determine which lesions “lend themselves to reproducible repeated measurements”. This is also sustained in our study, where the removal of rank causes the correlation between the feature importances of the models of the two radiologists to drop significantly. Besides rank features, size-related features, namely volume, 2D and 3D diameters, were among the features with the highest importances, albeit distinctly lower than those of rank. The reliance on size has been reported by Morse et al. [14], who suggested that readers often tend to prioritize selecting the largest tumors, regardless of the reproducibility of their measurement. This tendency disregards the recommended practice of choosing lesions that allow for reproducible repeated measurements, even if they are not the largest.

Overall, similarly to the selection of measurable lesions, we observed a considerable disagreement between the two readers on target lesion selection. It is worth noting that since target lesion selection happens after measurable lesion selection, the results of the first influence the second. In light of this, we observed that in nearly all patients with measurable lesions agreement, there was also target lesion agreement, which highlights the importance of agreement on measurability from the beginning. Nevertheless, caution should be taken in assuming that achieving a good consensus on measurability leads to a good consensus on selecting target lesions, as other factors could be at play. For example, patients with higher measurable lesion agreement had fewer lesions to start with, making target lesion selection disagreement less likely, even if lesions were chosen at random. Additionally, other factors, such as the prospective memory previously discussed, may also be influencing the selection process.

Target lesion selection has been appointed as the major source of variability when applying RECIST. Tovoli et al. [16] found that the choice of target lesions was the primary factor leading to disagreement among skilled readers in hepatocellular carcinoma, while less skilled readers disagreed more on lesion measurement. A study by Kuhl et al. [10] revealed poor agreement among radiologists in selecting target lesions in patients with metastatic disease. This was attributed to the apparent similar suitability that different lesions can have as targets, meaning that different sites of metastasis seem equally suitable, resulting in inconsistent assessments of treatment response. In ovarian cancer, Krasovitsky et al. [9] found high levels of lesion selection reproducibility subject to the location of the lesions, where pelvic mass lesions showed the highest reproducibility and lower for peritoneal metastases — although this should be framed in the context of their study design, where a relatively small total number of lesions per patient (maximum six) was analyzed, thus minimizing the chances of disagreement. Nonetheless, Krasovitsky warned about the risks of the assumption that there is “radiological truth” in response assessment. This lack of this radiological truth further raises questions regarding the reliability and validity of the label “radiological progression by RECIST” which still plays a pivotal role in defining surrogate endpoints such as progression-free survival in clinical trials and drug testing [15].

The most significant limitation of this study is the modest, retrospective dataset, coupled with a limited number of readers involved. Extrapolating these results to a larger cohort needs validation. Furthermore, when interpreting these results, one must take into account that, while readers are in practice limited to a maximum of five target lesions in total (and no more than two per organ) per patient, the models trained here do not have that restriction. Further modeling could include awareness of the models to the patient level, as well as the already selected lesions. This point is also relevant when accounting for the analysis of the performance. All results are split between overall (all lesions) and patient-level (all lesions of each individual patient). However, the statistical comparison between the two is not straightforward, due to grouped nature of the data, and to the requirements of the statistical metric (ROC-AUC) — in practical terms, it is not be possible, for example, to compute the ROC-AUC when all lesions in single patient were all measurable. Even though the likelihood of lesion selection disagreements occurring due to measurement errors is discussed, we did not conduct any further analysis on measurement errors by, for example, asking the readers to perform independent segmentations or to report their measurement, or by checking the concordance between the measured diameter and the ground truth extracted by the segmentation of the lesion. Another important aspect of lesion selection that was not analyzed was reader experience, relying on the assumption that all trained radiologists are equally likely to be asked by study coordinators to perform RECIST measurements.

## 5 Conclusion

This study leveraged machine learning to explore the lesions’ characteristics influencing the selection of measurable and target lesions based on RECIST criteria. Our models successfully predicted the selection of measurable and target lesions, with size- and rank-related features being critical in the decision process of individual radiologists, respectively. Nevertheless, substantial disagreement was still observed between radiologists, particularly in the selection of measurable and target lesions, likely attributed to ambiguous guidelines, reliance on individual experience, and the interaction of the consecutive tasks of measurable and target lesion selection. Our study highlights the need for refined guidelines to achieve better agreement on measurability and lesion selection that can overcome the potential influence of individual, subjective reading patterns.

## Ethical statement for solid state ionics

Hereby, I /insert author name/ consciously assure that for the manuscript /insert title/ the following is fulfilled:

1) This material is the authors' own original work, which has not been previously published elsewhere.
2) The paper is not currently being considered for publication elsewhere.
3) The paper reflects the authors' own research and analysis in a truthful and complete manner.
4) The paper properly credits the meaningful contributions of co-authors and co-researchers.
5) The results are appropriately placed in the context of prior and existing research.
6) All sources used are properly disclosed (correct citation). Literally copying of text must be indicated as such by using quotation marks and giving proper reference.
7) All authors have been personally and actively involved in substantial work leading to the paper, and will take public responsibility for its content.

## Funding

This study has no external funding sources to declare.

## CRediT authorship contribution statement

**Liliana Petrychenko:** Writing – review & editing, Writing – original draft, Validation, Data curation, Conceptualization. **Teresa M Tareco Bucho:** Writing – review & editing, Writing – original draft, Visualization, Validation, Software, Methodology, Investigation, Formal analysis, Data curation, Conceptualization. **Mohamed Abdelatty:** Writing – review & editing, Methodology, Investigation, Data curation. **Stefano Trebeschi:** Writing – review & editing, Writing – original draft, Validation, Supervision, Resources, Project administration, Methodology, Investigation, Conceptualization. **Regina Beets-Tan:** Resources, Project administration, Funding acquisition. **Zuhir Bodalal:** Writing – review & editing, Data curation. **Nino Bogveradze:** Writing – review & editing, Data curation.

## Declaration of Competing Interest

The authors declare that they have no known competing financial interests or personal relationships that could have appeared to influence the work reported in this paper.
