# Supplementary material for: Reproducing RECIST lesion selection via machine learning: Insights into intra and inter-radiologist variation
Source: Eur J Radiol Open. 2024 Apr 17;12:100562. doi: 10.1016/j.ejro.2024.100562 (PMC11039940; doi:10.1016/j.ejro.2024.100562)

# SUPPLEMENT 1

PROTOCOL OF THE (PANCANCER) STUDY FOR LESION SEGMENTATION

1. **Segmentation process**
   1. Read the report - estimate lesions to be segmented
   2. Load nrrd file to 3D slicer - straight from the shared drive
   3. Identify lesions to be segmented.
      1. If you are not confident to identify lesion → refer to PACS
      2. If still not sure after looking into clinical data → save it for discussion
   4. Segment all malignant lesion identified - separate segmentation label for each lesion
   5. Label each segmentation by numbers (codes) as following:

0 - Primary tumor (use 0.1; 0.2 etc. in case there are two primary tumours)

1 - Lymph nodes (use 1.1; 1.2; 1.3 etc. for multiple suspicious lymph nodes)

2 - Lung/Pleural mets (use 2.1; 2.2; 2.3 etc. for multiple lung/pleural mets)

3 - Liver mets (use 3.1; 3.2; 3.3 etc. for multiple liver mets)

4 - Abdopelvis deposits (use 4.1; 4.2; 4.3 etc. for multiple deposits)

5 - Bone mets (use 5.1; 5.2; 5.3 etc. for multiple bone mets)

6 - Brain mets (use 6.1; 6.2; 6.3 etc for multiple brain mets)

7 - Adrenal mets (use 7.1; 7.2; 7.3 etc. for several other lesions)

8 - Others (use 8.1; 8.2; 8.3 etc. for several other lesions)

- 1. Save segmentation to the same folder with original images (make sure to save the Scene too, just in case)
  2. The Excel document (in your shared drive) has an empty column next to location biopsy and the translation - the name of the column is “biopsy segments”. Fill the cell with segmentation code (numbers) corresponding to the location of biopsy, if possible. Sometimes it is clear (for example from lung primary tumor, liver solitary mets etc). BUT if it is not clear (for example lymph nodes without exact location, liver mets without clear location) fill the cell with segmentation code with possible biopsy location (can be multiple, please specify all).

1. **Specific situations**
   1. Multiple lymph nodes (LNs)
      1. Please segment all nodes that are highly suspicious of malignancy.
      2. If there are multiple LNs in 1 region, or if you are unsure of the malignancy of the LN
         1. Please use 1cm (short diameter) cut off
         2. Segment all LNs that are over 1cm in short diameter
   2. Multiple metastasis
      1. If there are numerous metastases, please segment a minimum of 10 lesions (largest) in 1 organ. (Please start with most significant lesions)
      2. If you are sure all of them are feasible for segmentation, please segment them all.
   3. Conglomerated or hard to ‘define’ lesions
      1. Use your best judgment to separately segment them. You can always discuss in your group.
      2. In case of surrounding tumor or internal fluid component,
         1. Try not to segment fluid content (both inside and outside)
         2. If it is difficult to separate, especially within the tumor, it is ok to include them. Please use your best judgement and skills.

# SUPPLEMENT 2

List of radiomic features fed to the random forest models. Rank, position, and lesion vicinity features not included. A short description of the size features is provided to facilitate interpretation of the results. All other features descriptions can be found in the original documentation of the library PyRadiomics.

| **Feature Name** | **Feature Description** |
| --- | --- |
| original_shape_Elongation | Elongation shows the relationship between the two largest principal components in the ROI shape. |
| original_shape_Flatness | Flatness shows the relationship between the largest and smallest principal components in the ROI shape. |
| original_shape_LeastAxisLength | This feature yield the smallest axis length of the ROI-enclosing ellipsoid |
| original_shape_MajorAxisLength | Major axis=4√(λ_major_) \| This feature yield the largest axis length of the ROI-enclosing ellipsoid and is calculated using the largest principal component λ_major_ |
| original_shape_Maximum2DDiameterColumn | Maximum 2D diameter (Column) is defined as the largest pairwise Euclidean distance between tumor surface mesh vertices in the row-slice (usually the **coronal**) plane. |
| original_shape_Maximum2DDiameterRow | Maximum 2D diameter (Row) is defined as the largest pairwise Euclidean distance between tumor surface mesh vertices in the column-slice (usually the **sagittal**) plane. |
| original_shape_Maximum2DDiameterSlice | Maximum 2D diameter (Slice) is defined as the largest pairwise Euclidean distance between tumor surface mesh vertices in the row-column (generally the **axial**) plane. |
| original_shape_Maximum3DDiameter | Maximum 3D diameter is defined as the largest pairwise Euclidean distance between tumor surface mesh vertices. |
| original_shape_MeshVolume | The volume of the ROI is calculated from the triangle mesh of the ROI. For each face in the mesh the (signed) volume of the tetrahedron defined by that face and the origin of the image is calculated. |
| original_shape_MinorAxisLength | minor axis=4√(λ_minor_) \| This feature yield the second-largest axis length of the ROI-enclosing ellipsoid and is calculated using the largest principal component λ_minor_ |
| original_shape_Sphericity | Sphericity is a measure of the roundness of the shape of the tumor region relative to a sphere |
| original_shape_SurfaceArea | To calculate the surface area, first the surface area of each triangle in the mesh is calculated. The total surface area is then obtained by taking the sum of all calculated sub-areas. |
| original_shape_SurfaceVolumeRatio | A lower value indicates a more compact (sphere-like) shape. This feature is not dimensionless, and is therefore (partly) dependent on the volume of the ROI. |
| original_shape_VoxelVolume | The volume of the ROI is approximated by multiplying the number of voxels in the ROI by the volume of a single voxel |
| original_firstorder_10Percentile |  |
| original_firstorder_90Percentile |  |
| original_firstorder_Energy |  |
| original_firstorder_Entropy |  |
| original_firstorder_InterquartileRange |  |
| original_firstorder_Kurtosis |  |
| original_firstorder_Maximum |  |
| original_firstorder_MeanAbsoluteDeviation |  |
| original_firstorder_Mean |  |
| original_firstorder_Median |  |
| original_firstorder_Minimum |  |
| original_firstorder_Range |  |
| original_firstorder_RobustMeanAbsoluteDeviation |  |
| original_firstorder_RootMeanSquared |  |
| original_firstorder_Skewness |  |
| original_firstorder_TotalEnergy |  |
| original_firstorder_Uniformity |  |
| original_firstorder_Variance |  |
| original_glcm_Autocorrelation |  |
| original_glcm_ClusterProminence |  |
| original_glcm_ClusterShade |  |
| original_glcm_ClusterTendency |  |
| original_glcm_Contrast |  |
| original_glcm_Correlation |  |
| original_glcm_DifferenceAverage |  |
| original_glcm_DifferenceEntropy |  |
| original_glcm_DifferenceVariance |  |
| original_glcm_Id |  |
| original_glcm_Idm |  |
| original_glcm_Idmn |  |
| original_glcm_Idn |  |
| original_glcm_Imc1 |  |
| original_glcm_Imc2 |  |
| original_glcm_InverseVariance |  |
| original_glcm_JointAverage |  |
| original_glcm_JointEnergy |  |
| original_glcm_JointEntropy |  |
| original_glcm_MCC |  |
| original_glcm_MaximumProbability |  |
| original_glcm_SumAverage |  |
| original_glcm_SumEntropy |  |
| original_glcm_SumSquares |  |

#

# SUPPLEMENT 3

Code to reproduce the experiment, explained step-by-step.

Dataset format

| **PatientID** | **LesionID** | **Organ** | **Measurable_R1** | **Target_R1** | **Measurable_R2** | **Target_R2** |
| --- | --- | --- | --- | --- | --- | --- |
| Pt_0001 | 1 | Lung | Yes | Yes | Yes | Yes |
| Pt_0002 | 1 | Liver | Yes | Yes | Yes | No |
| Pt_0002 | 2 | Liver | Yes | No | No | No |

*Step 1 - Define a feature extraction procedure*

| from radiomics import featureextractor  extractor = featureextractor.RadiomicsFeatureExtractor()  extractor.disableAllFeatures()  extractor.enableFeatureClassByName('firstorder')  extractor.enableFeatureClassByName('glcm')  extractor.enableFeatureClassByName('shape') |
| --- |

*Step 2 - Extract features from each single lesion*

| from radiomics import imageoperations  from SimpleITK import ReadImage as imread  from SimpleITK import GetArrayFromImage as im2arr  # loop records  for index, row in dataset.iterrows():  im, mask = imread(im_path), imread(mask_path)  im, mask = im2arr(im), im2arr(mask)  # bb is the bounding box, upon which the image and mask are cropped  bbImage, bbMask = imageoperations.checkMask(image, mask, label=label)  croppedImage, croppedMask = imageoperations.cropToTumorMask(image, mask, bbImage)  # get radiomics features  features_dict = extractor.execute(croppedImage, croppedMask) |
| --- |

*Step 3 - Extract rank and lesion vicinity*

| import numpy as np  from help import get_centroids  from help import get_distance  from help import get_size  def compute_distance_nearest_lesion(mask, lesion):  centroid = get_centroid(mask, lesion)  # all other lesions  all_lesions = np.unique(mask)[1:]  all_lesions.remove(lesion)  all_centroids = [get_centroid(mask, l) for l in all_lesions]    # distances to all other lesions  distances = [get_distance(centroid, c) for c in all_centroids]  return min(distance)  def compute_label_rank(mask, lesion):  unique_labels, label_counts = np.unique(mask, return_counts=True)  label_sizes = label_counts[unique_labels == label]  # Remove background label (usually 0)  unique_labels = unique_labels[1:]  label_counts = label_counts[1:]  # Sort sizes in descending order  sorted_sizes = np.sort(label_sizes)[::-1]  # Compute rank  rank = np.where(sorted_sizes == label_sizes)[0][0] + 1    return rank  # loop records  for index, row in dataset.iterrows():  im, mask = imread(im_path), imread(mask_path)  im, mask = im2arr(im), im2arr(mask)  nearest = compute_distance_nearest_lesion(mask, lesion)  rank = compute_label_rank(mask, lesion) |
| --- |

*Step 4 - Train random forest classifier to predict whether a lesion is selected (target or measurable)*

| from sklearn.ensemble import RandomForestClassifier  from help_functions import PatientShuffleSplit  # Fixing the seed for the RF (≠ Monte Carlo seed)  fixed_seed = 42  # number of monte carlo cross validation splits  MonteCarloCVLoops = 100  for seed in range(MonteCarloCVLoops):  # split into train-test (70-30) on a patient level  # X contains lesion features, y contains the Measurable/Target outcome  X_train, X_test, y_train, y_test, _ , ts_pats = \  PatientShuffleSplit(X, seed, obj, rtrn_names = True)  # train  model = RandomForestClassifier(random_state = fixed_seed)  model.fit(X_train, y_train)  # evaluate  y_pred = model.predict_proba(X_test)  auc = roc_auc_score(y_test, y_pred)  auc_patient = roc_auc_score(y_test, y_pred, patient_id)  feature_imp = model.feature_importance_   log_results(auc, auc_patient, feature_imp) |
| --- |

*Step 5 - Statistical analysis*

| import numpy as np  import pandas as pd  from scipy import stats  from help_functions import eliminate_nans  def statistic(x, y, axis):  return np.median(x, axis=axis) - np.median(y, axis=axis)  def permutations_test(group_data1, group_data2):  # scipy.stats.permutation_test  # permutation_type = 'samples' : observations are assigned to different samples but remain  # paired with the same observations from other samples.  # This permutation type is appropriate for paired sample hypothesis tests  # such as the Wilcoxon signed-rank test and the paired t-test.  # removing pairs of the monte-carlo where the AUC was nan in at least one of them  eliminate_nans = pd.DataFrame([list(group_data1), list(group_data2)]).T  eliminate_nans.columns=['group1','group2']  eliminate_nans = eliminate_nans.dropna(subset=['group1','group2'], how='any')  a = stats.permutation_test(  (eliminate_nans['group1'], eliminate_nans['group2']),  statistic,  vectorized=True,  random_state = 123,  permutation_type = 'samples',  n_resamples=100000,  alternative='two-sided'  )  return a |
| --- |

#

# SUPPLEMENT 4

Distribution of segmented lesions per organ, for N=40 patients.

| **Total** | 263 |
| --- | --- |
| **Liver** | 88 |
| **Mediastinal Lymph Nodes** | 42 |
| **Lung** | 40 |
| **Abdominal** | 34 |
| **Bone** | 25 |
| **Abdominal Lymph Nodes** | 13 |
| **Neck Lymph Nodes** | 11 |
| **Adrenal** | 4 |
| **Axillary Lymph Nodes** | 3 |
| **Pelvis** | 2 |
| **Esophagus** | 1 |

#

# SUPPLEMENT 5

Top 5 features with the highest mean accuracy decrease for both readers in measurable and target lesion prediction, with no ablation of features (None), ablation of diameter and correlated features (Diameter),ablation of diameter, volume and correlated features (Diameter + Volume) and ablation of ranks and correlated features (Ranks).

| **Ablation** | **Reader** | **Feature 1** | **Feature 2** | **Feature 3** | **Feature 4** | **Feature 5** |
| --- | --- | --- | --- | --- | --- | --- |
| *Predicting measurable lesions* | | | | | | |
| **None** | 1 | Surface Volume Ratio | Minor Axis Length | Voxel Volume | Least Axis Length | Mesh Volume |
|  | 2 | Surface Volume Ratio | Minor Axis Length | Voxel Volume | Mesh Volume | Least Axis Length |
| **Diameter** | 1 | Voxel Volume | Kurtosis | Overall Rank | Range | Minimum |
|  | 2 | Voxel Volume | Range | Minimum | Kurtosis | Overall Rank |
| **Diameter + Volume** | 1 | Range | Minimum | Overall Rank | Kurtosis | Distance closest lesion |
|  | 2 | Range | Minimum | Overall Rank | Kurtosis | Autocorrelation |
| **Ranks** | 1 | IDMN | IDN | Range | Distance closest lesion | Kurtosis |
|  | 2 | IDMN | IDN | Range | Minimum | Maximum |
| *Predicting target lesions* | | | | | | |
| **None** | 1 | Organ Rank | Overall Rank | Voxel Volume | Maximum 2D Diameter Column | Maximum 3D Diameter |
|  | 2 | Organ Rank | Overall Rank | Major Axis Length | Maximum 3D Diameter | Voxel Volume |
| **Diameter** | 1 | Overall Rank | Organ Rank | Voxel Volume | Root Mean Squared | Organ |
|  | 2 | Organ Rank | Overall Rank | Voxel Volume | Flatness | MCC |
| **Diameter + Volume** | 1 | Organ Rank | Overall Rank | Root Mean Squared | Organ | Range |
|  | 2 | Organ Rank | Overall Rank | Sphericity | Flatness | Skewness |
| **Ranks** | 1 | Maximum | IDMN | Flatness | IDN | Cluster Shade |
|  | 2 | Sphericity | Flatness | MCC | Elongation | IDN |

# SUPPLEMENT 6


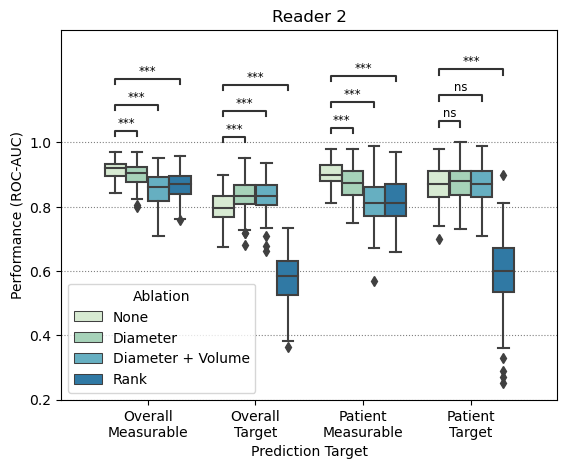

Supplement: Supplementary file 1 — Supplementary material [file mmc1.docx]
